# MONO, DI and TRI SSRs data extraction & storage from 1403 virus genomes with next generation retrieval mechanism

**DOI:** 10.1016/j.dib.2017.06.008

**Published:** 2017-06-10

**Authors:** K.V.S.S.R. Murthy, K.V.V. Satyanarayana

**Affiliations:** a Department of CSE, SRKR Engineering College, Bhimavaram, AP 534204, India; b Department of CSE, K L University, Vaddeswaram, Guntur, AP 522502, India

## Abstract

Now a day׳s SSRs occupy the dominant role in different areas of bio-informatics like new virus identification, DNA finger printing, paternity & maternity identification, disease identification, future disease expectations and possibilities etc., Due to their wide applications in various fields and their significance, SSRs have been the area of interest for many researchers. In the SSRs extraction, retrieval algorithms are used; if retrieval algorithms quality is improved then automatically SSRs extraction system will achieve the most relevant results. For this retrieval purpose in this paper a new retrieval mechanism is proposed which will extracted the MONO, DI and TRI patterns. To extract the MONO, DI and TRI patterns using proposed retrieval mechanism in this paper, DNA sequence of 1403 virus genome data sets are considered and different MONO, DI and TRI patterns are searched in the data genome sequence file. The proposed Next Generation Sequencing (NGS) retrieval mechanism extracted the MONO, DI and TRI patterns without missing anything. It is observed that the retrieval mechanism reduces the unnecessary comparisons. Finally the extracted SSRs provide the useful, single view and useful resource to researchers.

**Specifications Table**

**Table:** 

Subject area	Bio-informatics
More specific subject area	Genomes of VIRUSES
Type of data	Tables, figures
How data was acquired	VIRUS SSR markers extraction with NGS string matching
Data format	Analyzed
Experimental factors	MONO, DI and TRI SSRs: *A,C,G,T,AC,AG,…,ACC,…*were targeted. NGS retrieval process is applied on genomes VIRUSES. MONO, DI and TRI SSR markers to be used in various detection purposes are extracted with this approach.
Experimental features	Each of the MONO, DI and TRI markers are extracted from genomes of VIRUSES. All the SSRs showed the 1,2,3-bp in allele size. These differences showed that there are some polymorphisms among the genomes to the number of SSR repeats.
Data source location	BHIMAVARAM, INDIA
Data accessibility	The data is provided with this article

**Value of the data**

• Data sets obtained from genomes of VIRUSES with NGS retrieval process have shown the high specificity.
• These data suggest that SSR extraction is an useful method for providing information for various applications related to studies in VIRUSES.
• Access to the raw sequencing data in VIRUSES allows researchers to perform further bio-informatics analysis based on their own computational algorithms.

## 1 Data

**Fig. 1:**
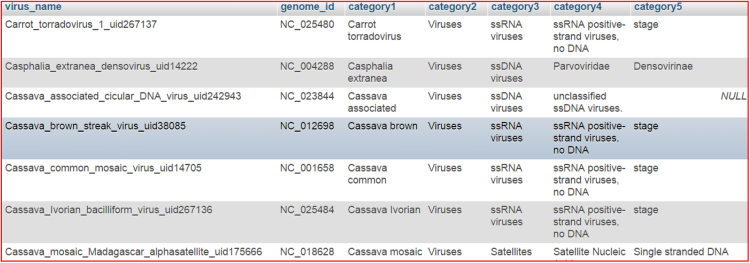
virus_category table actual data.

Database has been developed using MySQL. The information stored in the database includes virus names, genome id, A,C,G,T percentages, tract length, category, motif types (MONO, DI and TRI), the sequences of the motifs and frequencies of occurrence in the entire genome. The actual process of database is shown in Fig. 1.

### 1.1 Structure of the database

In this paper, we consider three tables from database and changed the structure to our own format so that additional analysis can be done easily. They are

1. virus_category
2. virus_acgt_count
3. virus_ssrs

#### 1.1.1 Virus category

This table has the information related to virus categories from virus files. The structure is as shown in the Table 1 and actual data was shown in Fig. 1.

**Table 1:** virus_category.

Type	Collation
virus_name	varchar(100)
genome_id	varchar(20)
category1	varchar(20)
category2	varchar(20)
–	–

#### 1.1.2 Virus ACGT count

**Fig. 2:**
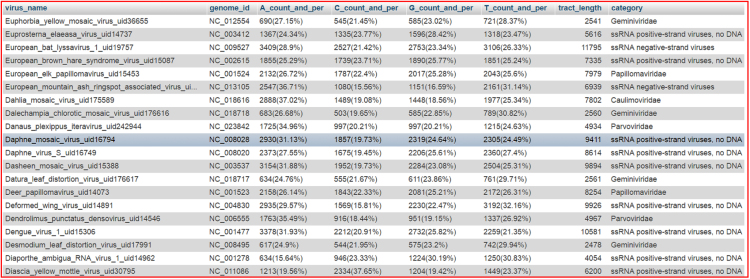
virus_acgt_count table actual data.

This table has the information related to virus A,C,G and T count, its percentage, tract length. The structure is as shown in the Table 2 and actual data was shown in Fig. 2.

**Table 2:** virus_category.

Type	Collation
**virus_name**	varchar(100)
**genome_id**	varchar(20)
**A_count_and_per**	varchar(20)
**C_count_and_per**	varchar(20)
**G_count_and_per**	varchar(20)
**T_count_and_per**	varchar(20)
**tract_length**	int(15)
**category**	varchar(20)

#### 1.1.3 Virus SSRs

**Fig. 3:**
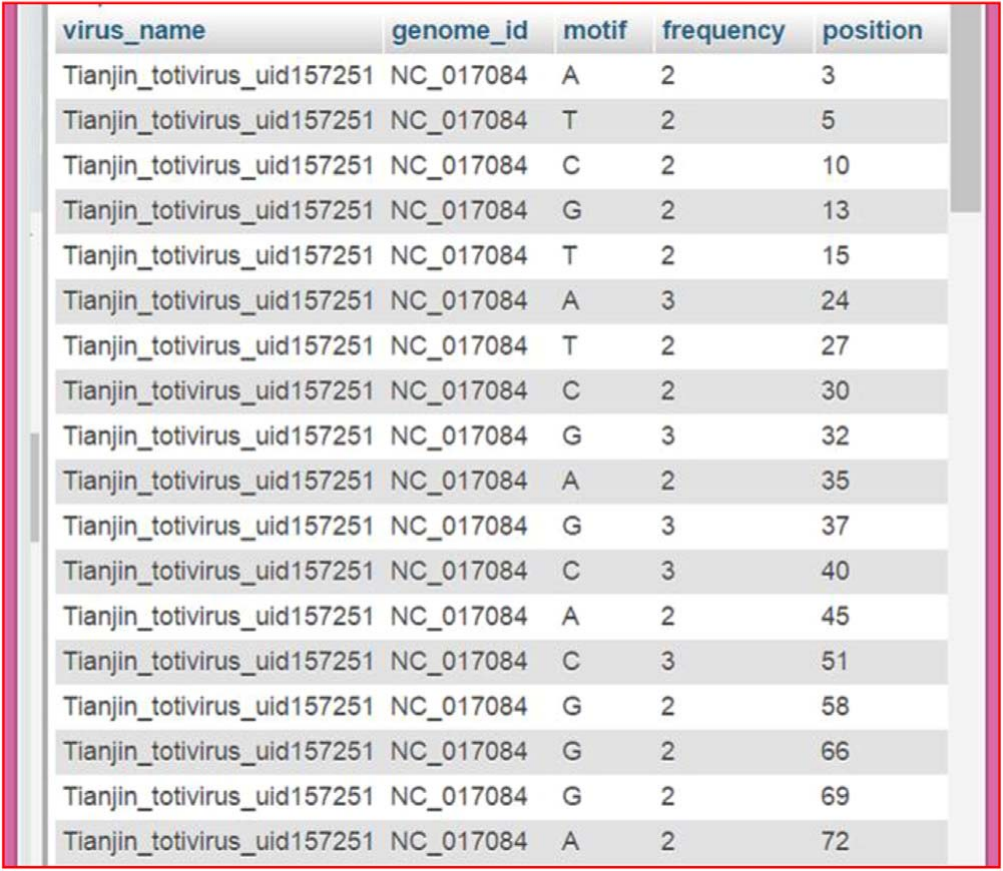
virus_ssrs table actual data.

This table has the information related to virus_name, genome_id, motif, frequency and its position. The structure is as shown in the Table 3 and actual data was shown in Fig. 3.

**Table 3:** virus_ssrs.

Type	Collation
**virus_name**	varchar(100)
**genome_id**	varchar(20)
**motif**	varchar(20)
**frequency**	int(10)
**position**	int(10)

### 1.2 Description

In this section we give detailed description of the 1403 virus genomes

#### 1.2.1 Category wise description

**Fig. 4:**
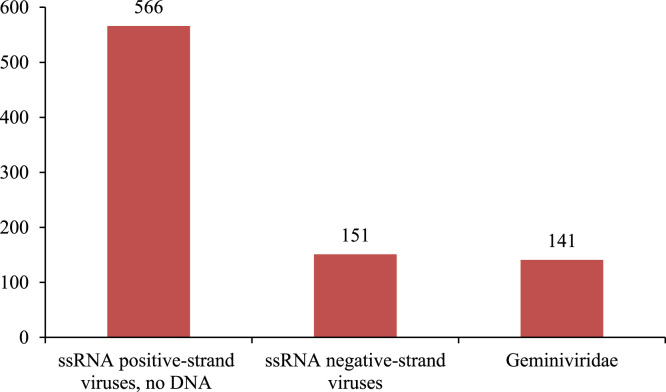
category wise virus count.

We used a total of 1403 virus genome sequences. We categorized these genomes as shown in the Table A1(presented in Appendix A). From this categorization (according to Table A1), we observe that virus genomes are further sub grouped into 49 categories. They are Amalgaviridae, Ampullaviridae, Anelloviridae etc., Among the 1403 genomes, 566 genomes belong to ssRNA positive-strand viruses, no DNA, 151 belong to ssRNA negative-strand viruses, 141 belong to Geminiviridae etc.,. From the Fig. 4, observed that ssRNA positive-strand viruses, no DNA (566), ssRNA negative-strand viruses (151), Geminiviridae (141) occupies the major role among the others.

#### 1.2.2 Frequency description

We extracted the overall frequency, MONO, DI and TRI frequencies from the virus_ssrs those are shown in Table 4. From these extracted information MONO has shown the max frequency that is 99, so it has high impact.

**Table 4:** Virus genome overall frequency, MONO, DI and TRI frequencies.

**FREQUENCY**			
	**MIN**	**AVG**	**MAX**
**OVERALL**	**1**	1.2482250811894526	**99**
**MONO**	**10**	2.4448562907955393	**99**
**DI**	**1**	1.0749041913092998	**9**
**TRI**	**1**	1.0247784693226274	**9**

#### 1.2.3 Virus size description

In this section, we described SSRs by executing SQL queries on virus_category for category wise counts and the results are shown in the Table A2 (presented in Appendix A). Table A2 gives a summary of the total number of genomes categorized based on genome sizes of various virus categories. Two of the Mimiviridae genomes are found to be very high (greater than 1 Mb), 81 ssRNA negative-strand viruses and 89 ssRNA positive-strand viruses, no DNA are found to be between the 10 Kb and 50 Kb. 31 virus genomes have shown size less than <1 Kb.

#### 1.2.4 MIN, MAX and AVG tract length description

**Fig. 5:**
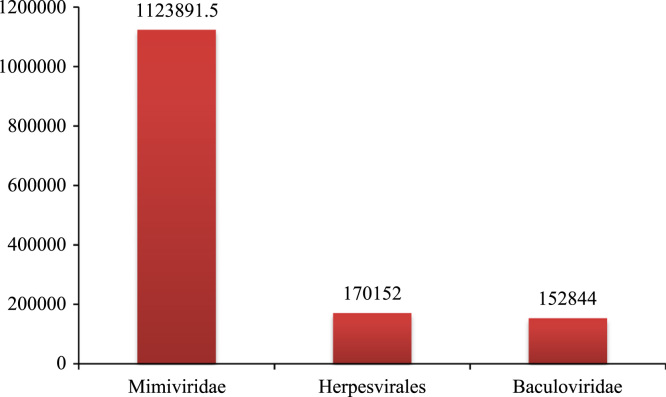
average tract length analysis.

We did a preliminary study on the genome sizes of all viruses as shown in the Table A3 (presented in Appendix A). From the Table A3, we observed that, the smallest Mitochondrial genome is Satellite Nucleic Acids of length 216 bp whereas the largest virus genome is Mimiviridae of length 1,241,026 bp. When the average genome sizes of viruses are considered with respect to their category, it has been observed that the average lengths of Mimiviridae genomes are much higher when compared to those of Herpesvirales and Baculoviridae (Refer Fig. 5). The virus genomes of Mimiviridae are around 6 times larger than those of Herpesvirales and 7 times larger than Baculoviridae genomes.

#### 1.2.5 MONO MOTIF description

We extract the total of 4,692,149 continues MONO, DI and TRI SSRs are extracted from 1403 genomes. Table A4 (presented in Appendix A) shown the max frequency of the MONO motifs.

#### 1.2.6 DI MOTIF description

We extract a total of 12853740 continues DI SSRs are extracted from 1403 genomes. Table A5(presented in Appendix A) shown the max frequency of the DI motifs.

#### 1.2.7 TRI MOTIF description

We extract a total of 14469215 continues TRI SSRs are extracted from 1403 genomes. Table A6(presented in Appendix A) shown the max frequency of the TRI motifs.

## 2 Experimental design, materials and methods

### 2.1 SSR extraction

Availability of next-generation sequencing techniques leads to the accessibility of genome sequences including that of organelles like virus, fungi, bacteria etc. Studying the hyper-mutating SSRs [1], [2], [3], [4], [5], [6] repeats in virus genomes using Bioinformatics approach would be very interesting and informative as SSRs mining not only helps in understanding and addressing biological questions but also helps in making the best use of these repeats in various diverse applications. Earlier, few studies have attempted to analyze the distribution of SSR repeats in virus genomes but they are confined to a single or a small set of genomes. So far, there are no comprehensive reports in literature that show the distribution of microsatellite repeats in all sequenced virus genomes. In the remaining part of this study, we analyzed SSR repeats in more than 1403 virus genomes and a brief note on the distribution and frequency of these repeats has been presented.

**Fig. 6:**
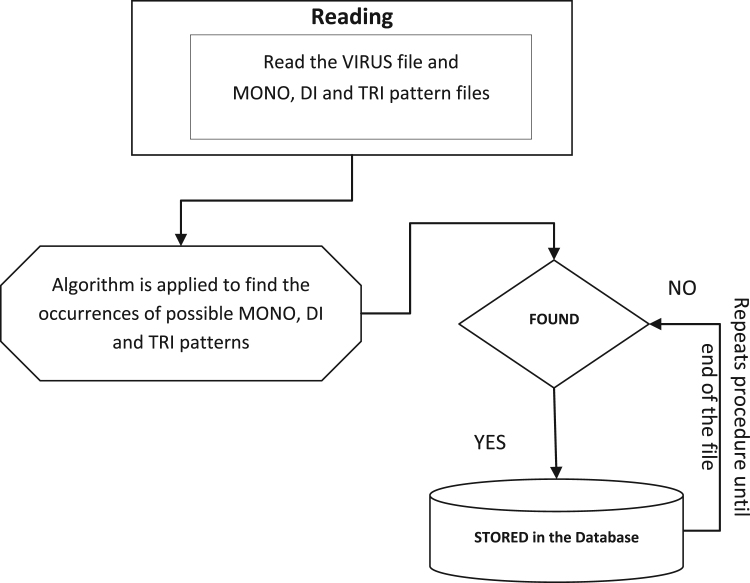
MONO,DI & TRI extraction process.

This approach scans the input virus genome sequence file and pattern files for MONO, DI and TRI patterns to find all occurrences of these patterns within this file using next generation retrieval mechanisms [7], [8], [9]. If repeat occurs then the successive logic is applied. The successive logic means continuous occurrence of similar patterns. If the successive pattern size >1 then the successive occurrence of pattern information is stored in the database. The process is shown in Fig. 6. The database is constructed in MySQL using JAVA.

SSR NGS retrieval algorithm has shown the detailed explanation about the Next Generation Sequencing(NGS) retrieval algorithm. It consists of five segments called I/O, Main, search, tandem repeat checking and database insertion. In input segment virus and pattern files are considered as input. In output segment, the extracted mechanism provides the number of occurrences, positions of MONO, DI and TRI patterns. In Main segment the length of file and pattern are read, for each pattern, *ngs_search, check_for_tandem_repeat and ngs_database_insertion* segments are called for entire length of input file. In search segment, the pattern is searched in the input file, if match occurs then increments the occurrence count. In tandem repeat checking segment, the different between the occurrence positions are measured, if they are equal to length of the pattern then it is considered one tandem repeat. In database insertion segment, virus name, genome id, pattern, count and position is stored in the database.

**Table:** 

		**SSR NGS RETRIEVAL ALGORITHM**
**Input:***Virus files and MONO, DI and TRI pattern files* **Output:***The number of occurrences and the positions of the MONO, DI and TRI pattern*		
/* Main */		
1	n←T.length, m←P.length	
2	for each MONO, DI & TRI patterns	
3	for i ← 0 to n-m do	
4	begin	
5	count←ngs_search(T,P,i,count);	
6	tandem_repeat_count←check_for_tandem_repeat(T,P,i,count);	
7	ngs_database_insertion(P,i,tandem_repeat_count)	
8	end for	
9	end for	
/* Search */		
18	*int ngs_search(Char[] T, Char[] P, int i, int count)*	
19	begin	
20	$j_{1}$← P.length;	
21	while ( $j_{1}$>=0 && T[ i - $j_{1}$] == P[$j_{1}$])	
22	do	
23	$j_{1}$←$j_{1}$-1;	
24	done;	
25	if ($j_{1}$== -1)	
26	count++;	
27	end if	
28	return count;	
29	*end ngs_search;*	
/* Tandem repeat checking */		
30	*int check_for_tandem_repeat(Char[] T, Char[] P, int i, int count)*	
31	begin	
32	if (diff_of_two_repeats==-P.length)	
33	tandem_repeat_count++;	
34	else	
35	tandem_repeat_count= tandem_repeat_count;	
36	end if	
37	return tandem_repeat_count;	
38	*end check_for_tandem_repeat;*	
39	/* Database insertion */	
40	*ngs_database_insertion(Char[] P, int i, int tandem_repeat_count)*	
41	begin	
42	insert into virus_ssrs(virus_name, genome_id, P, tandem_repeat_count,i);	
43	*end ngs_database_insertion;*	

## Figures and Tables

**Table A1:** Category wise virus genome sequences.

**CATEGORY**	**COUNT**
Amalgaviridae	4
Ampullaviridae	1
Anelloviridae	6
Aumaivirus.	1
Bacilladnavirus	4
Baculoviridae	1
Bicaudaviridae	1
Birnaviridae	4
Botybirnavirus.	1
Caudovirales	14
Caulimoviridae	34
Chrysoviridae	2
Circoviridae	35
Corticoviridae	1
Endornaviridae	8
Fuselloviridae	4
Geminiviridae	141
Hepadnaviridae	10
Herpesvirales	2
Hypoviridae	3
Inoviridae	7
Lavidaviridae	1
Ligamenvirales	6
Microviridae	5
Mimiviridae	2
Nanoviridae	5
Papanivirus.	1
Papillomaviridae	85
Partitiviridae	21
Parvoviridae	40
Polyomaviridae	39
Poxviridae	1
Reoviridae	3
Retroviridae	42
Salterprovirus	2
Satellite Nucleic Acids	75
Satellites	4
ssRNA negative-strand viruses	151
ssRNA positive-strand viruses, no DNA	566
Totiviridae	26
Turriviridae	1
unassigned ssRNA viruses	1
unclassified dsDNA phages.	1
unclassified dsDNA viruses.	2
unclassified Gemycircularvirus.	7
unclassified ssDNA viruses.	30
unclassified ssRNA viruses.	2
Total	1403

**Table A2:** Virus genome sizes and their classification based on different size ranges.

**Genome size range**	**No. of genomes**
**SIZE < 1 Kb**	
**CATEGORY**	**COUNT**
Circoviridae	1
Nanoviridae	3
Papanivirus.	1
Partitiviridae	2
Satellite Nucleic Acids	20
ssRNA negative-strand viruses	2
ssRNA positive-strand viruses, no DNA	2
**>=1 Kb and <2 Kb**	
**Category**	**Count**
Aumaivirus.	1
Circoviridae	27
Nanoviridae	2
Partitiviridae	12
Reoviridae	1
Satellite Nucleic Acids	55
Satellites	4
ssRNA negative-strand viruses	15
ssRNA positive-strand viruses, no DNA	12
unclassified ssDNA viruses.	6
**>=10 Kb < 50 Kb**	
**Category**	**Count**
Ampullaviridae	1
Caudovirales	8
Endornaviridae	7
Fuselloviridae	4
Hypoviridae	1
Lavidaviridae	1
Ligamenvirales	6
Retroviridae	8
Salterprovirus	2
ssRNA negative-strand viruses	81
ssRNA positive-strand viruses, no DNA	89
Totiviridae	1
Turriviridae	1
unclassified dsDNA viruses.	1
unclassified ssDNA viruses.	1
**>=50 Kb < 100 Kb**	
**Category**	**Count**
Bicaudaviridae	1
Caudovirales	2
**>=100 Kb < 500 Kb**	
**Category**	**Count**
Baculoviridae	1
Caudovirales	3
Herpesvirales	2
Poxviridae	1
**Size > 1 Mb**	
**Category**	**Count**
Mimiviridae	2

**Table A3:** Virus ggenome sizes of Mitochondria category wise.

**Category**	**Smallest**	**Largest**	**Average**
Amalgaviridae	3110	3387	3314.0000
Ampullaviridae	23471	23471	23,471.0000
Anelloviridae	2109	3720	2782.8333
Aumaivirus.	1151	1151	1151.0000
Bacilladnavirus	5472	5914	5668.2500
Baculoviridae	152844	152844	152,844.0000
Bicaudaviridae	61833	61833	61,833.0000
Birnaviridae	2744	3380	3203.5000
Botybirnavirus.	6126	6126	6126.0000
Caudovirales	7203	165318	58,854.2857
Caulimoviridae	6845	9073	7683.9706
Chrysoviridae	2860	3203	3031.5000
Circoviridae	846	2883	1920.8286
Corticoviridae	9935	9935	9935.0000
Endornaviridae	9620	17236	13,734.1250
Fuselloviridae	14634	23840	17,159.0000
Geminiviridae	2456	3588	2664.9504
Hepadnaviridae	2974	3328	3115.7000
Herpesvirales	131808	208496	170,152.0000
Hypoviridae	9406	12552	10,526.0000
Inoviridae	5721	8339	6957.4286
Lavidaviridae	17029	17029	17,029.0000
Ligamenvirales	24302	40582	36,293.8333
Microviridae	4070	6360	5200.4000
Mimiviridae	1006757	1241026	1,123,891.5000
Nanoviridae	965	1083	1010.2000
null	928	9877	4157.1429
Papanivirus.	814	814	814.0000
Papillomaviridae	6919	8484	7556.4353
Partitiviridae	303	2315	1730.7143
Parvoviridae	3726	6243	5048.6000
Polyomaviridae	4629	6130	5056.7692
Poxviridae	142509	142509	142,509.0000
Reoviridae	1646	2752	2333.0000
Retroviridae	3120	13056	8384.5238
Salterprovirus	14255	15837	15,046.0000
Satellite Nucleic Acids	216	1457	1127.6133
Satellites	1326	1342	1335.2500
ssRNA negative-strand viruses	800	18688	8945.1523
ssRNA positive-strand viruses, no DNA	944	19901	7476.2845
Totiviridae	2066	11394	5663.6538
Turriviridae	16382	16382	16,382.0000
unassigned ssRNA viruses	4312	4312	4312.0000
unclassified dsDNA phages.	8059	8059	8059.0000
unclassified dsDNA viruses.	7966	14914	11,440.0000
unclassified Gemycircularvirus.	2059	2218	2139.1429
unclassified ssDNA viruses.	1788	10503	3369.4333
unclassified ssRNA viruses.	5916	6195	6055.5000

**Table A4:** **MONO SSRs**.

**VIRUS_NAME**	**genome_id**	**MOTIF**	**MAX FREQUENCY**	**Number of times occurred**
Feline_astrovirus_2_uid218014	NC_022249	G	99	1
		**A**	**9**	**313**
Abalone_herpesvirus_Victoria_AUS_2009_uid177933	NC_018874	A	9	3
Eupatorium_yellow_vein_virus_satellite_DNA_beta_ui…	NC_004515	A	9	3
Hedyotis_uncinella_yellow_mosaic_betasatellite_uid…	NC_023015	A	9	2
Honeysuckle_yellow_vein_mosaic_disease_associated_…	NC_009571	A	9	2
Malvastrum_yellow_mosaic_virus_satellite_DNA_beta_…	NC_008560	A	9	2
Mamestra_configurata_NPV_A_uid14168	NC_003529	A	9	4
Megavirus_chiliensis_uid74349	NC_016072	A	9	118
Moumouvirus_uid186430	NC_020104	A	9	71
		**C**	**9**	**57**
Abalone_herpesvirus_Victoria_AUS_2009_uid177933	NC_018874	C	9	2
Canine_papillomavirus___4_uid28243	NC_010226	C	9	2
Feline_leukemia_virus_uid14686	NC_001940	C	9	7
Potato_mop_top_virus_uid14789	NC_003723	C	9	3
Tolypocladium_cylindrosporum_virus_1_uid61451	NC_014823	C	9	2
Trichechus_manatus_latirostris_papillomavirus_2_ui…	NC_016898	C	9	2
		**T**	**9**	**268**
Trematomus_polyomavirus_1_uid282773	NC_026944	T	9	2
Canine_oral_papillomavirus_uid14326	NC_001619	T	9	2
Chaetoceros_lorenzianus_DNA_Virus_uid63565	NC_015211	T	9	2
Citrus_chlorotic_dwarf_associated_virus_uid170854	NC_018151	T	9	2
Ferret_papillomavirus_uid218024	NC_022253	T	9	2
Megavirus_chiliensis_uid74349	NC_016072	T	9	115
Mamestra_configurata_NPV_A_uid14168	NC_003529	T	9	4
Moumouvirus_uid186430	NC_020104	T	9	78
Abalone_herpesvirus_Victoria_AUS_2009_uid177933	NC_018874	T	9	2

**Table A5:** **DI SSRs**.

**VIRUS_NAME**	**genome_id**	**MOTIF**	**MAX FREQUENCY**	**Number of times occurred**
		AC	9	**1**
Sauropus_leaf_curl_disease_associated_DNA_beta_uid…	NC_018671	AC	9	**1**
		AG	7	1
Vanilla_distortion_mosaic_virus_uid263828	NC_025250	AG	7	1
		AT	9	2
Moumouvirus_uid186430	NC_020104	AT	9	2
Zalophus_californianus_papillomavirus_1_uid65277	NC_015325	CG	7	1
		CT	7	3
Baboon_endogenous_virus_M7_uid222253	NC_022517	CT	7	2
Cowpea_mosaic_virus_uid15283	NC_003549	CT	7	1
		CA	9	1
Sauropus_leaf_curl_disease_associated_DNA_beta_uid…	NC_018671	CA	9	1
		GT	8	3
Spleen_focus_forming_virus_uid14641	NC_001500	GT	8	1
Norway_rat_hepacivirus_1_uid267736	NC_025672	GT	8	1
Human_papillomavirus_type_26_uid15507	NC_001583	GT	8	1
		GA	6	2
Vanilla_distortion_mosaic_virus_uid263828	NC_025250	GA	6	1
Oat_golden_stripe_virus_uid15093	NC_002358	GA	6	1
		GC	6	1
Zalophus_californianus_papillomavirus_1_uid65277	NC_015325	GC	6	1
		TA	9	1
Moumouvirus_uid186430	NC_020104	TA	9	1
		TC	7	1
Cowpea_mosaic_virus_uid15283	NC_003549	TC	7	1
		TG	NULL	NULL

**Table A6:** TRI SSRs.

**VIRUS_NAME**	**genome_id**	**MOTIF**	**MAX FREQUENCY**	**Number of times occurred**
		AAC	7	**1**
Penicillium_chrysogenum_virus_uid16141	NC_007540	AAC	7	**1**
Santeuil_nodavirus_uid62547	NC_015069	AAG	6	**1**
Mamestra_configurata_NPV_A_uid14168	NC_003529	AAT	7	**1**
Penicillium_chrysogenum_virus_uid16141	NC_007540	ACA	7	**1**
		ACC	4	**16**
Zamilon_virophage_uid230580	NC_022990	ACC	4	**1**
–				
Human_papillomavirus_type_49_uid15455	NC_001591	ACC	4	**1**
Mamestra_configurata_NPV_A_uid14168	NC_003529	ACG	5	**1**
Microviridae_phi_CA82_uid70009	NC_015785	ACT	6	**1**
Santeuil_nodavirus_uid62547	NC_015069	AGA	7	**1**
Ursus_maritimus_papillomavirus_1_uid29915	NC_010739	AGC	6	**1**
		AGG	6	**4**
Procyon_lotor_papillomavirus_1_uid15468	NC_007150	AGG	6	**1**
–				
Epsilonpapillomavirus_1_uid14220	NC_004195	AGG	6	**1**
		AGT	6	**1**
Mamestra_configurata_NPV_A_uid14168	NC_003529	AGT	6	**1**
Abalone_herpesvirus_Victoria_AUS_2009_uid177933	NC_018874	AGT	6	**1**
		AGT	6	**2**
Mamestra_configurata_NPV_A_uid14168	NC_003529	ATA	6	**1**
Himetobi_P_virus_uid14801	NC_003782	ATA	6	**1**
Mamestra_configurata_NPV_A_uid14168	NC_003529	ATC	9	**1**
		ATG	5	**2**
Potato_yellow_dwarf_virus_uid74995	NC_016136	ATG	5	**1**
Puumala_virus_uid14930	NC_005225	ATG	5	**1**
		ATT	4	**11**
Mamestra_configurata_NPV_A_uid14168	NC_003529	ATT	4	**3**
–				
		CAA	6	**2**
Penicillium_chrysogenum_virus_uid16141	NC_007540	CAA	6	**1**
Cucumber_green_mottle_mosaic_virus_uid14681	NC_001801	CAA	6	**1**
		CAC	4	**9**
Zamilon_virophage_uid230580	NC_022990	CAC	4	**1**
–				
Magnaporthe_oryzae_chrysovirus_1_uid51685	NC_014465	CAC	4	**1**
		CAG	6	**3**
Ursus_maritimus_papillomavirus_1_uid29915	NC_010739	CAG	6	**1**
–				
Mamestra_configurata_NPV_A_uid14168	NC_003529	CAG	6	**1**
Mamestra_configurata_NPV_A_uid14168	NC_003529	CAT	8	**1**
Mamestra_configurata_NPV_A_uid14168	NC_003529	CAT	8	**1**
		CCA	4	**13**
Zamilon_virophage_uid230580	NC_022990	CCA	4	**1**
–				
Abalone_herpesvirus_Victoria_AUS_2009_uid177933	NC_018874	CCA	4	**1**
		CCG	4	**5**
Phlebiopsis_gigantea_mycovirus_dsRNA_1_uid46855	NC_013999	CCG	4	**1**
–				
Halastavi_arva_RNA_virus_uid77939	NC_016418	CCG	4	**1**
		CCT	6	**3**
Curionopolis_virus_uid264939	NC_025354	CCT	6	**1**
–				
Abalone_herpesvirus_Victoria_AUS_2009_uid177933	NC_018874	CCT	6	**1**
		CGA	5	**2**
Mamestra_configurata_NPV_A_uid14168	NC_003529	CGA	5	**1**
Human_papillomavirus_109_uid36519	NC_012485	CGA	5	**1**
		CGC	4	**9**
Phlebiopsis_gigantea_mycovirus_dsRNA_1_uid46855	NC_013999	CGC	4	**1**
–				
Horseshoe_bat_hepatitis_B_virus_uid253463	NC_024444	CGC	4	**1**
		CGG	4	**6**
Woolly_monkey_sarcoma_virus_uid19547	NC_009424	CGG	4	**1**
–				
Abalone_herpesvirus_Victoria_AUS_2009_uid177933	NC_018874	CGG	4	**1**
Mamestra_configurata_NPV_A_uid14168	NC_003529	CGT	6	**1**
Microviridae_phi_CA82_uid70009	NC_015785	CTA	6	**1**
Mamestra_configurata_NPV_A_uid14168	NC_003529	CTC	7	**1**
		CTG	4	**9**
Saguaro_cactus_virus_uid14981	NC_001780	CTG	4	**1**
–				
Abalone_herpesvirus_Victoria_AUS_2009_uid177933	NC_018874	CTG	4	**1**
Abalone_herpesvirus_Victoria_AUS_2009_uid177933	NC_018874	CTT	7	**1**
Santeuil_nodavirus_uid62547	NC_015069	GAA	6	**1**
		GAC	5	**2**
Mamestra_configurata_NPV_A_uid14168	NC_003529	GAC	5	**2**
		GAG	7	**3**
Procyon_lotor_papillomavirus_1_uid15468	NC_007150	GAG	7	**1**
–				
Crocuta_papillomavirus_1_uid174774	NC_018575	GAG	7	**1**
		GAT	5	**2**
Puumala_virus_uid14930	NC_005225	GAT	5	
Acidianus_bottle_shaped_virus_uid19605	NC_009452	GAT	5	
Ursus_maritimus_papillomavirus_1_uid29915	NC_010739	GCA	7	**1**
		GCC	4	**7**
Raphanus_sativus_cryptic_virus_1_uid17127	NC_008190	GCC	4	**1**
–				
Mycobacteriophage_Velveteen_uid215123	NC_022060	GCC	4	**1**
Halorubrum_pleomorphic_virus_3_uid157259	NC_017088	GCG	5	**1**
		GCT	5	**3**
Saguaro_cactus_virus_uid14981	NC_001780	GCT	5	**1**
–				
Mamestra_configurata_NPV_A_uid14168	NC_003529	GCT	5	**1**
		GGA	6	**5**
Procyon_lotor_papillomavirus_1_uid15468	NC_007150	GGA	6	**1**
–				
Human_papillomavirus_type_103_uid17119	NC_008188	GGA	6	**1**
Halorubrum_pleomorphic_virus_3_uid157259	NC_017088	GGC	4	**1**
Mamestra_configurata_NPV_A_uid14168	NC_003529	GGT	5	**1**
Mamestra_configurata_NPV_A_uid14168	NC_003529	GTC	6	**1**
		GTG	4	**7**
Periplaneta_fuliginosa_densovirus_uid14091	NC_000936	GTG	4	**1**
–				
Mamestra_configurata_NPV_A_uid14168	NC_003529	GTG	4	**1**
		GTT	5	**3**
Cherry_rasp_leaf_virus_uid15131	NC_006271	GTT	5	**1**
–				
Ovine_enzootic_nasal_tumour_virus_uid15410	NC_007015	GTT	5	**1**
		TAA	6	**2**
Mamestra_configurata_NPV_A_uid14168	NC_003529	TAA	6	**1**
Himetobi_P_virus_uid14801	NC_003782	TAA	6	**1**
Microviridae_phi_CA82_uid70009	NC_015785	TAC	6	**1**
Mamestra_configurata_NPV_A_uid14168	NC_003529	TAG	5	**1**
		TAT	4	**9**
Yaba_like_disease_virus_uid14595	NC_002642	TAT	4	**1**
–				
Human_papillomavirus_54_uid15466	NC_001676	TAT	4	**1**
Mamestra_configurata_NPV_A_uid14168	NC_003529	TCA	8	**1**
		TCC	6	**4**
Curionopolis_virus_uid264939	NC_025354	TCC	6	**1**
–				
Mamestra_configurata_NPV_A_uid14168	NC_003529	TCC	6	**1**
Mamestra_configurata_NPV_A_uid14168	NC_003529	TCG	6	**1**
		TCT	5	**3**
Mamestra_configurata_NPV_A_uid14168	NC_003529	TCT	5	**1**
–				
Nyamanini_virus_uid38109	NC_012703	TCT	5	**1**
		TGA	5	**2**
Puumala_virus_uid14930	NC_005225	TGA	5	**1**
Cycas_necrotic_stunt_virus_uid15397	NC_003791	TGA	5	**1**
		TGC	5	**2**
Chicken_gallivirus_1_uid259980	NC_024770	TGC	5	**1**
Mamestra_configurata_NPV_A_uid14168	NC_003529	TGC	5	**1**
		TGG	4	**12**
Peanut_clump_virus_uid14776	NC_003668	TGG	4	**1**
–				
Acinetobacter_bacteriophage_AP22_uid167576	NC_017984	TGG	4	**1**
		TGT	5	**2**
Cherry_rasp_leaf_virus_uid15131	NC_006271	TGT	5	**1**
Ovine_enzootic_nasal_tumour_virus_uid15410	NC_007015	TGT	5	**1**
		TTA	5	**2**
Walleye_dermal_sarcoma_virus_uid14718	NC_001867	TTA	5	**1**
Mamestra_configurata_NPV_A_uid14168	NC_003529	TTA	5	**1**
		TTC	5	**4**
Squash_leaf_curl_China_virus____B__uid15591	NC_007339	TTC	5	**1**
–				
Nyamanini_virus_uid38109	NC_012703	TTC	5	**1**
		TTG	NULL	

## Appendix A

See Table A1, Table A2, Table A3, Table A4, Table A5, Table A6 here
