# Supplementary material for: MONO, DI and TRI SSRs data extraction & storage from 1403 virus genomes with next generation retrieval mechanism
Source: Data Brief. 2017 Jun 10;13:326–40. doi: 10.1016/j.dib.2017.06.008 (PMC5476967; doi:10.1016/j.dib.2017.06.008)
Supplement: Supplementary file 1 — Supplementary material [file mmc1.docx]

Authors’ not having conflict of interest
